# Real-world clinical outcomes among US Veterans with oral factor xa inhibitor–related major bleeding treated with andexanet alfa or 4-factor prothrombin complex concentrate

**DOI:** 10.1007/s11239-023-02820-y

**Published:** 2023-05-23

**Authors:** S. Scott Sutton, Joseph Magagnoli, Tammy H. Cummings, Theresa Dettling, Belinda Lovelace, Mary J. Christoph, James W. Hardin

**Affiliations:** 1 Dorn Research Institute, Columbia VA Healthcare System (151), 6439 Garners Ferry Road, Columbia, SC 29209 USA; 2 Department of Clinical Pharmacy and Outcomes Sciences, College of Pharmacy, University of South Carolina, Columbia, SC USA; 3 Global Health Economics and Outcomes Research, AstraZeneca Rare Disease, Alexion, USA; 4 Department of Epidemiology and Biostatistics, University of South Carolina, 6439 Garners Ferry Road, Columbia, SC 29209 USA

**Keywords:** Andexanet alfa, 4F-PCC, Bleed, Mortality, Veteran

## Abstract

Oral factor Xa (FXa) inhibitors significantly reduce incidence of stroke and thromboembolic events in patients with atrial fibrillation or venous thromboembolism. Due to various factors and the lack of a randomized controlled trial comparing andexanet alfa to usual care, non-specific replacement agents including 4 F-PCC are still used off-label for FXa inhibitor bleed management. Clinical and mortality data were extracted from the inpatient medical data and Veteran Affairs (VA) vital status files over the time of March 2014 through December 2020. Propensity score-weighted models were used for this retrospective cohort study using data from the Veterans Affairs Informatics and Computing Infrastructure (VINCI). The study included 255 patients (85-andexanet alfa and 170-4 F-PCC) exposed to an oral factor Xa inhibitor and hospitalized with an acute major, gastrointestinal (GI), intracranial (ICH) or other bleed. In-hospital mortality was significantly lower in the andexanet alfa cohort compared to the 4 F-PCC cohort (10.6% vs. 25.3%, p = 0.01). Propensity score–weighted Cox models reveal a 69% lower hazard of in-hospital mortality for those treated with andexanet alfa (HR 0.31, 95% CI 0.14–0.71) compared to those treated with 4 F-PCC. Additionally, those treated with andexanet alfa had a lower 30-day mortality rate and lower 30-day hazard of mortality in the weighted Cox model (20.0% vs. 32.4%, p = 0.039; HR 0.54, 95% CI 0.30–0.98) compared to those treated with 4 F-PCC. Among 255 US veterans with major bleeding in the presence of an oral factor Xa inhibitor, treatment with andexanet alfa was associated with lower in-hospital and 30-day mortality than treatment with 4 F-PCC.

**Supplementary Information:**

The online version contains supplementary material available at 10.1007/s11239-023-02820-y.

## Highlights

There is a lack of a randomized controlled trial comparing andexanet alfa to usual care, non-specific replacement agents including 4 F-PCC are still used off-label for FXa inhibitor bleed management.

Among US veterans with major bleeding in the presence of an oral factor Xa inhibitor, treatment with andexanet alfa was associated with lower in-hospital and 30-day mortality than treatment with 4 F-PCC.

Further research is needed to confirm the results of this analysis and better understand the real-world clinical outcomes associated with use of andexanet alfa and 4 F-PCC in the presence of oral FXa inhibitor-related bleeding and regarding bleeding outcomes, especially around baseline bleed severity and long-term functional status after the bleeding event.

## Introduction

Oral factor Xa (FXa) inhibitors significantly reduce incidence of stroke and thromboembolic events in patients with atrial fibrillation or venous thromboembolism [1–3]. Additionally, compared to warfarin, FXa inhibitors have comparable efficacy and improved safety profiles [4, 5]. Specifically, regarding safety, the incidence of intracranial hemorrhage (ICH) in patients receiving FXa inhibitors within trial settings is 0.1–0.3% compared to 0.3–0.6% for warfarin [6]. Although FXa inhibitors have lower bleeding rates, the potential for bleeding complications exists and there is a critical need for treatment options to restore hemostasis, prevent further expansion [7], and reduce mortality associated with bleeding events not related to anticoagulants [8–10].

Prior to 2018, 4-factor prothrombin complex concentrate (4 F-PCC) was commonly used off-label for management of rivaroxaban- and apixaban-related bleeds due to the lack of specific reversal agent. Additionally, although protamine sulfate is recommended to neutralize enoxaparin-related bleeding or overdose [11], 4 F-PCC has also been utilized in attempt to manage enoxaparin-related bleeds. Since US Food and Drug Administration (FDA) approval in 2018, the specific reversal agent coagulation factor Xa (recombinant) inactivated-zhzo (USAN andexanet alfa) has been available to manage life-threatening or uncontrolled bleeding associated with rivaroxaban and apixaban [12]. However, due to factors including initial availability, cost, and the lack of a randomized controlled trial comparing andexanet alfa to usual care, non-specific factor replacement including 4 F-PCC are still used off-label for FXa inhibitor-related bleed management despite patients not having factor depletion. Factor replacement therapy including some 4 F-PCC products could also potentially have off-target effects due to additional components of heparin and proteins C and S [13]. Although patients with major bleeding in the ANNEXA-4 trial (ClinicalTrials.gov Identifier: NCT02329327) had rapid decreases in anti-FXa activity, hemostatic efficacy rates of 80%, and 30-day mortality rates of 15.7% [14], additional data are needed due to the single-arm nature of the trial. Randomized clinical trials are needed to clarify the optimal agent for reversal of factor Xa inhibitors, and the ongoing ANNEXA-I trial (NCT03661528) will address this gap among patients with intracranial hemorrhage. In the interim, it is critical to perform comparative effectiveness studies among real-world populations across multiple bleed types.

Therefore, we aimed to conduct a real-world retrospective analysis assessing clinical outcomes among United States Veterans treated with andexanet alfa compared to 4 F-PCC for the management of major bleeding in the presence of rivaroxaban, apixaban, edoxaban or enoxaparin.

## Materials and methods

### Database

This retrospective cohort study used data from the US Department of Veterans Affairs. Individual-level information on demographics, administrative claims, and pharmacy dispensation were obtained from the Veterans Affairs Informatics and Computing Infrastructure (VINCI). The completeness, utility, accuracy, validity, and access methods are described on the VA website, http://www.virec.research.va.gov (search term VINCI).

### Permission

The study was conducted in compliance with the Department of Veterans Affairs requirements and received Institutional Review Board (IRB) and Research and Development approval (Dorn Research Institute, Columbia VA Healthcare System; IRB#1,573,544; approval date 6/19/2020; study title: Burden of illness for oral factor Xa inhibitors (OFXai) related bleeds). Informed consent was waived for this retrospective non-observational study for the Dorn Research Institute IRB. The study utilized inpatient and outpatient data consisting of claims coded with International Classification of Diseases (ICD) revision 9, 10-CM, Current Procedure Terminology (CPT) as well as pharmacy, laboratory, and vital sign data from March 2014 to December 2020.

### Cohort selection

Patients were included if they (1) received andexanet alfa or 4 F-PCC infusion over the time period 2014 to December 2020 during an inpatient or hospital-based encounter based extracted from the VA intravenous (IV) medication data, (2) had an ICD-9-CM or ICD-10-CM code indicating either a gastrointestinal (GI), intracranial hemorrhage (ICH) or other bleed based on bleeding codes (Table S1) [15, 16], (3) received a prescription at any time point prior to index hospitalization for apixaban, rivaroxaban, edoxaban or enoxaparin (either inpatient or outpatient) (4) had complete hospital admission and discharge data, and (5) were adults age 18 or older. Patients were excluded if they had a prescription dispensed for warfarin or dabigatran within the previous 90 days of the bleed diagnosis.

### Study outcome

The primary outcomes for the study were in-hospital and 30-day mortality. In-hospital mortality was defined by a discharge disposition coded as deceased. Thirty-day mortality was indicated if patients had a date of death within 30 days of andexanet alfa or 4 F-PCC administration. Dates of death data were extracted from the VA vital status files. Secondary outcomes included hospital length of stay (LOS), intensive care unit (ICU) length of stay, and discharge disposition.

### Baseline Data

Baseline demographic characteristics included age, race, and sex. Comorbid characteristics included composite indices such as the Charlson Comorbidity Index. Other clinical characteristics included bleed type (as coded from diagnosis codes), ventilation, invasive ventilation, and concomitant medications (including vitamin K, tranexamic acid, platelets, fresh frozen plasma, activated 4 F-PCC, and plasma cryoprecipitates). Ventilation and invasive ventilation were extracted using ICD-PCS,CPT codes. Concomitant medications were identified via procedure codes (ICD-PCS, CPT), barcode medication administration (BCMA) pharmacy files, and intravenous medication files.

### Statistical analysis

To analyze the association between factor Xa inhibitor reversal or replacement agents and mortality, baseline demographic, comorbid, and clinical characteristics were compared for the andexanet alfa cohort and the 4 F-PCC cohort. P-values from the chi-square or t-test along with the standardized difference were used to evaluate the differences among the cohorts. The standardized difference was calculated by subtracting the treatment means and then dividing by the pooled standard deviation. Unadjusted odds ratios were calculated to estimate the relationship between treatment with andexanet alfa or 4 F-PCC and outcomes including in-hospital and 30-day mortality and discharge home (vs. other locations).

As a retrospective clinical study, treatment assignment was not randomized. Inverse probability treatment weights (IPTW) were used to minimize potential bias from non-random treatment assignment. Logistic regression was utilized to calculate the propensity score and subsequently stabilized weights [17–19]. All baseline covariates discussed above were included in the propensity score model. Propensity score–weighted Kaplan-Meier survival curves were estimated for unadjusted results. We estimated multivariable, propensity score–weighted Cox proportional hazards models for both in-hospital and 30-day mortality. Hazard ratios (HR) and respective 95% confidence intervals (CI) were used to compare the hazard of in-hospital death and 30-day mortality between the andexanet alfa and 4 F-PCC cohorts.

To model hospital length of stay a generalized linear model (GLM) with a gamma distribution and log link was fit, which has been shown to be relatively robust to a variety of data generating processes [20, 21]. Length of stay ratios are used to summarize the difference between the cohorts.

## Results

A total of 255 patients were included in the study. Apixaban was the most utilized anticoagulant and the most common bleed type was gastrointestinal (Table 1). On average, patients were in their 7th decade of life, predominantly male, and had an average Charlson comorbidity index greater than 5. Among the 255 patients, 85 patients received andexanet alfa and 170 patients received 4 F-PCC to manage the bleeding episode. In addition to the reversal or replacement agents, many patients received concomitant medications to manage the bleed (Table 1). Prevalence of concomitant medications was similar except for vitamin K administration, which was lower in the andexanet alfa cohort (10.6% vs. 44.1%, p < 0.001) compared to the 4 F-PCC cohort.

**Table 1:** Baseline Demographic and Clinical Characteristics

*Baseline Characteristics*	*Andexanet alfa (N = 85)*	*4 F-PCC (N = 170)*	*p-value*	*Standardized difference*
Age, mean (SD)	76.1 (10.0)	71.8 (12.0)	0.004	0.381
Race, n (%)			0.005	
*Black*	9 (10.6%)	44 (25.9%)		0.377
*Other/unknown*	6 (7.1%)	< 5		0.242
*White*	70 (82.4%)	122 (71.8%)		0.245
Sex, n (%)			0.373	
*Female*	0 (0%)	< 5		0.189
*Male*	85 (100%)	166 (97.7%)		0.189
History of atrial fibrillation, n (%)	74 (87.1%)	124 (72.9%)	0.017	0.359
History of deep vein thrombosis, n (%)	13 (15.3%)	25 (14.7%)	1	0.016
Charlson comorbidity Index				
*Mean (SD)*	5.42 (3.28)	5.71 (3.35)	0.515	0.087
*Median (IQR)*	5 (3–7)	5 (3–8)	0.497	
Bleed type, n (%)			0.421	
*GI*	39 (45.9%)	90 (52.9%)		0.141
*ICH*	25 (29.4%)	49 (28.8%)		0.013
*Other*	21 (24.7%)	31 (18.2%)		0.161
Anticoagulant, n (%)			< 0.001	
*Apixaban*	67 (78.8%)	81 (47.7%)		0.632
*Edoxaban*	< 5	0 (0%)		0.188
*Enoxaparin*	< 5	64 (37.7%)		0.837
*Rivaroxaban*	16 (18.8%)	25 (14.7%)		0.112
Ventilation, n (%)	16 (18.8%)	49 (28.8%)	0.115	0.229
Invasive ventilation, n (%)	9 (10.6%)	34 (20%)	0.086	0.251
Concomitant Medications, n (%)				
*Plasma*	7 (8.2%)	26 (15.3%)	0.166	0.21
*Cryoprecipitates*	< 5	9 (5.3%)	0.21	0.212
*Transfusion*	7 (8.2%)	27 (15.9%)	0.134	0.225
*Tranexamic acid*	< 5	< 5	1	0.047
*Vitamin K*	9 (10.6%)	75 (44.1%)	< 0.001	0.713
*Platelets*	9 (10.6%)	20 (11.8%)	0.944	0.037
*Red blood cells*	28 (32.9%)	66 (38.8%)	0.435	0.122
*Activated 4 F-PCC*	6 (7.1%)	6 (3.5%)	0.347	0.167
Year, n (%)			< 0.001	
*2014–2016*	0 (0%)	32 (18.8%)		0.568
*2017–2018*	0 (0%)	58 (34.1%)		0.814
*2019–2020*	85 (100%)	80 (47.1%)		1.108

4 F-PCC = four-factor prothrombin complex concentrate, GI = gastrointestinal, ICH = intracranial hemorrhage, IQR = interquartile range, SD = standard deviation

### Primary outcomes: In-hospital and 30-day mortality

Unadjusted analysis revealed a statistically significantly lower in-hospital mortality rate for andexanet alfa compared to 4 F-PCC (10.6% vs. 25.3%, p = 0.01) resulting in 65% lower odds of in-hospital mortality for andexanet alfa (OR 0.35, 95% CI 0.15–0.73) (Table 2). Additionally, the andexanet alfa cohort had a lower 30-day mortality rate and lower odds of 30-day mortality (20.0% vs. 32.4%, p = 0.039; OR 0.52, 95% CI: 0.28–0.96; Table 2). Several factors may influence mortality rates, especially among patients with an active bleed; therefore, we utilized a Cox proportional hazards model to control for potential covariates. Multi-variable models revealed results that patients treated with andexanet alfa had a 66.1% lower hazard of in-hospital mortality compared to 4 F-PCC (aHR 0.34, 95% CI: 0.16–0.74; Table 3). Similarly, risk of 30-day mortality was 45.8% lower among patients treated with andexanet alfa compared to 4 F-PCC (aHR 0.54, 95% CI: 0.31–0.95; Table 3).

**Table 2:** Clinical and Hospitalization Bivariate Outcome Data

Mortality and Discharge	*Andexanet alfa*	*4 F-PCC*	*p-value*	*OR (95%CI)*
	*N = 85*	* N = 170*		
In-hospital mortality, n (%)	9 (10.6%)	43 (25.3%)	0.01	0.35 (0.15–0.73)
30-day mortality, n (%)^*a*^	17 (20%)	55 (32.4%)	0.039	0.52 (0.28–0.96)
Discharge disposition, n (%)				
*Home*	49 (57.7%)	82 (48.2%)	0.127	1.46 (0.87–2.50)^*b*^
*VA/Community nursing home*	17 (20%)	27 (15.9%)		
*VA domiciliary*	0 (0%)	< 5		
*Other hospital/medical center*	8 (9.4%)	14 (8.2%)		
*Died*	9 (10.6%)	43 (25.3%)		
*Missing*	< 5	< 5		
***Length of stay outcomes, full cohort***	***Andexanet alfa***	***4 F-PCC***	***p-value***	***OR (95%CI)***
	***N = 85***	*** N = 170***		
Hospital length of stay in days, mean (SD)	11.3 (18.2)	12.0 (17.6)	0.746	N/A
Hospital length of stay, median (IQR)	7 (3–10)	6 (2.3–14.8)	0.709	N/A
ICU length of stay, mean (SD)	4.2 (8.03)	3.9 (6.0)	0.743	N/A
ICU length of stay, median (IQR)	1 (0–4)	2 (0–5)	0.626	N/A
***Length of stay outcomes among those surviving hospitalization***	***Andexanet alfa***	***4 F-PCC***	***p-value***	***OR (95%CI)***
	***N = 76***	*** N = 127***		
Hospital length of stay in days, mean (SD)	9.6 (14.8)	13.6 (19.2)	0.097	N/A
Hospital length of stay, median (IQR)	6 (3–10)	7 (4–18)	0.255	N/A
ICU length of stay, mean (SD)	4.0 (7.7)	3.7 (4.8)	0.806	N/A
ICU length of stay, median (IQR)	1 (0–4)	2 (0–5)	0.262	N/A

p values for differences in means are based on t-tests, p values for medians are based on the Wilcoxon test, and p values for categorical variables are based on Chi-square tests

^*a*^30-day mortality is inclusive of in-hospital mortality, except for patients who died within the hospital outside of the 30-day follow-up

^*b*^OR reflects the odds of discharge to home, where the reference group is discharge to any other location (VA/community nursing home, VA domiciliary, other hospital/medical center, missing or died). The corresponding p-value for chi-square comparison of discharge home vs. not home: p = 0.209

4 F-PCC = four-factor prothrombin complex concentrate, CI = confidence interval, ICU = intensive care unit, IQR = interquartile range, OR = odds ratio, SD = standard deviation

**Table 3:** Risk of Mortality: Cox Proportional Hazards Model

	*In-hospital Mortality*	*30-day Mortality*
*Variable*	*HR (95% CI)*	*HR (95% CI)*
Andexanet alfa vs. 4 F-PCC (ref)	0.34 (0.16–0.74)	0.54 (0.31–0.95)
Age	1.01 (0.98–1.04)	1.02 (0.99–1.04)
White vs. non-white (ref)	1.33 (0.65–2.72)	1.07 (0.60–1.91)
Male vs. Female (ref)	0.13 (0.03–0.62)	0.25 (0.06–1.12)
Charlson Comorbidity Index	1.11 (1.02–1.20)	1.09 (1.02–1.16)
Bleed type: ICH vs. GI (ref)	1.02 (0.49–2.10)	1.20 (0.68–2.12)
Bleed type: other vs. GI (ref)	0.65 (0.30–1.42)	0.68 (0.35–1.32)
Ventilation vs. no ventilation (ref)	3.03 (1.58–5.80)	3.64 (2.14–6.18)
Transfusion vs. no transfusion (ref)	1.63 (0.83–3.17)	1.10 (0.59–2.04)

4 F-PCC = four-factor prothrombin complex concentrate, CI = confidence interval, GI = gastrointestinal; HR = hazard ratio, ICH = intracranial hemorrhage, ref = reference group

Sample characteristics adjusted using IPTW weighting are shown in Table S2. IPTW weighted Kaplan-Meier survival curves for in-hospital mortality revealed a higher survival probability for patients treated with andexanet alfa compared to 4 F-PCC (Figure S1). The propensity score weighted Cox model revealed patients treated with andexanet alfa had a 69% lower risk of in-hospital death compared to 4 F-PCC (aHR 0.31, 95% CI: 0.14–0.71; Table 4). Survival curves for 30-day mortality demonstrated a similar trend (Figure S2), and weighted Cox model results found andexanet alfa treatment associated with a 45.6% lower risk of 30-day mortality compared to 4 F-PCC (aHR 0.54, 95% CI: 0.30–0.98; Table 4).

**Table 4:** Risk of Mortality: Propensity-weighted Cox Proportional Hazards Model

	*In-hospital Mortality*	*30-day Mortality*
*Variable*	*HR (95% CI)*	*HR (95% CI)*
Andexanet alfa vs. 4 F-PCC (ref)	0.31 (0.14–0.71)	0.54 (0.30–0.98)
Age	1.01 (0.98–1.05)	1.03 (0.99–1.06)
White vs. non-white (ref)	1.55 (0.73–3.30)	1.03 (0.57–1.86)
Male vs. Female (ref)	0.09 (0.02–0.36)	0.21 (0.03–1.65)
Charlson Comorbidity Index	1.14 (1.06–1.23)	1.10 (1.02–1.18)
Bleed type: ICH vs. GI (ref)	1.23 (0.57–2.67)	1.32 (0.70–2.47)
Bleed type: other vs. GI (ref)	0.57 (0.27–1.20)	0.43 (0.21–0.89)
Ventilation vs. no ventilation (ref)	3.53 (1.74–7.19)	5.74 (3.10-10.65)
Transfusion vs. no transfusion (ref)	1.72 (0.80–3.73)	1.12 (0.57–2.20)

4 F-PCC = four-factor prothrombin complex concentrate, CI = confidence interval, GI = gastrointestinal; HR = hazard ratio, ICH = intracranial hemorrhage, ref = reference group

### Secondary outcomes: discharge destination and length of Stay

Most patients (57.7%) treated with andexanet alfa were discharged to home, followed by 20% discharged to a VA or community nursing home, 10.6% died, 9.4% were transferred to another hospital or medical center, and 2 patients (2.2%) had missing discharge location data. Among patients treated with 4 F-PCC, 48.2% were discharged home, 25.3% died, 15.9% to a VA or community nursing home, 8.2% were transferred to another hospital or medical center, and 3 (1.76%) had missing discharge location data. Odds of being discharged home vs. another location other than home (including death or having missing discharge information) were higher for patients treated with andexanet alfa compared to those treated with 4 F-PCC; however, the difference was not statistically significant (OR 1.46, 95% CI: 0.87–2.50; Table 2).

Patients treated with andexanet alfa stayed in the hospital on average for 11.3 days compared to 12.0 days for 4 F-PCC (p = 0.746, Table 2); for the subset surviving the major bleed hospitalization, length of stay was on average 9.6 days for andexanet alfa and 13.6 days on average for 4 F-PCC (p = 0.097, Table 2). Median LOS was slightly longer for andexanet alfa (7 [IQR: 3–10] days) compared to 4 F-PCC (6 [IQR: 2.25–14.75] days) in the full population, and slightly shorter for andexanet alfa (6 [IQR: 3–10]) compared to 4 F-PCC (7 [IQR: 4–18]) among the subset of patients surviving the major bleed hospitalization. Adjusted model estimates revealed patients treated with andexanet alfa had a shorter average length of hospitalization compared to 4 F-PCC; however, consistent with initial results, the result was not statistically significant (LOS ratio 0.85, 95% CI 0.60–1.21; Table S3). The length of stay propensity weighted model demonstrated a shorter average length of stay for andexanet alfa compared to 4 F-PCC; however, the finding is not statistically significant (LOS ratio 0.88, 95% CI 0.64–1.23, Table S3).

Andexanet alfa-treated patients had a mean ICU length of stay of 4.2 days and 4 F-PCC treated patients had an average ICU length of stay of 3.9 days (Table 2). However, patients treated with andexanet alfa had a median ICU length of stay of 1 (IQR: 0–4) day compared to 2 (IQR: 0–5) days for 4 F-PCC (Table 2).

## Discussion

While FXa inhibitors have lower bleeding rates compared to warfarin, the risk for bleeding complications remains and there is a critical need for pharmacologic treatment options to reduce mortality associated with bleeding events. This study reports real-world effectiveness outcomes in a national cohort of patients managed with andexanet alfa or 4 F-PCC for a major bleed related to FXa inhibitor anticoagulants. Among 255 US veterans treated with andexanet alfa or 4 F-PCC for FXa inhibitor-related major bleeding, treatment with andexanet alfa was associated with significantly lower in-hospital and 30-day mortality. Patients treated with andexanet alfa also had a shorter median ICU length of stay; however, this finding was not statistically significant. The treatment of bleeding related to direct Factor Xa inhibitors including rivaroxaban, apixaban, and edoxaban and the indirect FXa inhibitor enoxaparin varies and historically has consisted of the off-label utilization of usual care agents such as 4 F-PCC, fresh frozen plasma, and vitamin K. Although 4 F-PCC is not FDA-indicated for management of active bleeds, observational studies have evaluated 4 F-PCC for treatment of bleeds related to rivaroxaban and apixaban [22–27]. These observational studies of varying methodologies and scientific rigor reported a 65–95% hemostatic efficacy rate and represented the primary treatment option until 2018. In 2018, andexanet alfa received conditional approval by the FDA under the accelerated approval pathway based on the results of the ANNEXA-A and ANNEXA-R clinical trials and the requirement that additional studies be conducted to demonstrate clinical outcomes [12]. In patients with acute major bleeding associated with the use of FXa inhibitors in the interim analysis of the single-arm ANNEXA-4 trial, treatment with andexanet alfa reduced anti-FXa activity and 80% of patients had excellent or good hemostatic efficacy at 12 h [14]. Additionally, andexanet alfa reversed the anticoagulant activity of apixaban and rivaroxaban at the end of the bolus administration and for the duration of the infusion [14].

Observational studies evaluated the real-world outcomes associated with use of andexanet alfa in patients with intracranial hemorrhage [28–30] and reported hemostatic effectiveness and mortality rates consistent with the ANNEXA-4 final study report analysis [14]. In a single-center retrospective case-series study evaluating 29 patients with an ICH that received either andexanet alfa (n = 18) or 4 F-PCC (n = 11), excellent-to-good hemostasis occurred in 88.9% of the andexanet-alfa patients compared to 60% of the 4 F-PCC cohort. In the andexanet alfa arm, in-hospital mortality was 12.5% (2/16) among patients with good/excellent hemostasis and 100% (2/2) among patients with poor hemostasis [28]. In the 4 F-PCC arm, in-hospital mortality was 50% (3/6) among patients with good/excellent hemostasis and 100% (4/4) among patients with poor hemostasis [28]. In another single-center comparative case series of patients with FXa inhibitor-related ICH, 30-day all-cause mortality was 30% among the 21 patients treated with andexanet alfa compared to 45.2% for the 35 patients treated with 4 F-PCC [29]. Additionally, a synthetic control arm study of 182 patients with intracerebral hemorrhage within the ANNEXA-4 trial and usual care patients within the RETRACE II registry in Germany showed that patients treated with andexanet alfa had lower risk of hematoma expansion (14% vs. 36% for usual care, adjusted relative risk: 0.40 [95% CI: 0.20–0.78, p = 0.005]). In-hospital mortality was 16.5% among patients in the andexanet alfa arm and 20.6% among patients treated with usual care, with an adjusted hazard ratio of 0.49 ([95%CI: 0.24–1.04], p = 0.06) [30].

In a real-world study examining electronic health records of 3,030 major bleeding events managed with andexanet alfa, 4 F-PCC, fresh frozen plasma, and other usual care agents, patients treated with andexanet alfa had a 4% in-hospital mortality rate of compared to 10% for four-factor prothrombin complex concentrate, 11% for fresh frozen plasma, and 8% for other agents or those who received no reversal, replacement, or hemostatic agents. Mortality also differed by bleed type, with patients who had intracranial hemorrhage experiencing higher mortality compared to GI, critical compartment, traumatic, or other bleed types. Notably though, reversal and replacement agent groups were not mutually exclusive and results were descriptive without adjustment for baseline severity [31].

### Strengths and Limitations

To our knowledge, our study is the largest direct comparison of clinical outcomes between patients treated with andexanet alfa or 4 F-PCC for FXa inhibitor-related bleeds. Furthermore, this study evaluated a national patient sample, utilizing electronic health records and pharmacy data among a large sample size consisting of a nationwide population. We studied patients in an integrated national healthcare system; therefore, the data are less susceptible to biases of single-center or regional studies. Additionally, this study evaluated multiple clinical outcomes including length of hospitalization, ICU length of stay, in-hospital and 30-day mortality, and discharge disposition, compared to previously published studies evaluating hemostatic effectiveness [32]. The addition of both 30-day mortality and discharge disposition are particularly key given that patients with major bleeding events related to anticoagulant use, particularly intracranial hemorrhage, may experience significant mortality outside of the hospitalization but before 30 days [33, 34], and/or poor functional outcomes [35]. Finally, we evaluated and adjusted for several demographic variables and comorbid conditions that are related to bleeding outcomes.

There are, however, limitations to our observational database analyses, particularly proper documentation, and coding. Since this study utilized an administrative claims database to evaluate mortality and length of stay, results are dependent upon accurate documentation and coding. Although our study evaluated important clinical factors (e.g., mortality), we could not evaluate functional status of the patients after treatment. Because this study was conducted utilizing administrative claims/codes, we could not evaluate medication dosage data (neither FXai dosage nor andexanet alfa or 4 F-PCC dosage) or patient disability via a tool such as the Modified Rankin Score (mRS). Furthermore, there are several patient level factors that could impact the interpretation and application of the results that were not evaluated in this study, including measurement of hematoma expansion and timing variables including time since last FXai dose and time from symptom onset to reversal or replacement administration, advance directives, thrombotic complications, and total cost. The inability to capture time since last dose of FXai in particular is a key limitation, as we cannot confirm the anticoagulation status of patients at the time of the bleeding event. Despite covariate adjustment and propensity weighting for relevant patient factors, we cannot rule out the possibility of selection bias or residual confounding. Notably, all statistical methods, including propensity score analysis have limitations. Ultimately propensity score weighting should balance both cohorts, minimizing the standardized difference between the groups. Figure S3 displays the boxplots for the standardized differences before and after weighting. While standardized differences decreased markedly, three covariates had standardized differences above 0.2 after-IPTW (e.g., Apixaban after-IPTW = 0.24, Enoxaparin after-IPTW = 0.39, Vitamin K after-IPTW = 0.33). A key assumption of the Cox model is proportional hazards over time. In the in-hospital mortality data, the IPTW-weighted Kaplan-Meier curves cross indicating non-proportionality. To investigate further, we undertook specification tests using accelerated failure time models (AFT) which do not assume proportionality, but however, do require proper specification of the baseline hazard. We fit models using the Weibull, exponential, log-normal, and log-logistic distributions. All results, presented in Table S4 reveal a statistically significant increase in survival times for andexanet alfa treated patients.

An additional study limitation is the population consisted primarily of white males in their 7th decade of life and included < 5 females among the 255 patients; therefore, our findings may not be generalizable to patients of different age or ethnic/racial groups or sex. Additionally, the treatments of interest were available over different time periods (with 4 F-PCC being available from 2014 to 2020 and andexanet alfa being available from 2019 to 2020). In a post-hoc sensitivity analysis including only patients treated between 2019 and 2020 when both treatments were available, results showed point estimates for the adjusted multivariable model were similar to the primary results for patients treated from 2014 to 2020, suggesting a lower mortality burden for patients treated with andexanet alfa. The study utilized data populated in VINCI and did not utilize registries, other data sets, or data from the Veterans Choice Program.

Notwithstanding the strengths and limitations, this study represents the first cohort study, to our knowledge, consisting of a national patient sample documenting the in-hospital and 30-day mortality for andexanet-alfa compared to 4 F-PCC among patients with FXa inhibitor-related bleeds.

## Conclusions

This study evaluated the real-world comparative effectiveness of andexanet alfa and 4 F-PCC utilizing a large, national claims and electronic health record database. In this sample, patients treated with andexanet alfa for FXa inhibitor-related bleeds had lower in-hospital and 30-day mortality rates compared to 4 F-PCC. Further research is needed regarding bleeding outcomes, especially around baseline bleed severity and long-term functional status after the bleeding event. Additional research is needed to confirm the results of this analysis and better understand the real-world clinical outcomes associated with use of andexanet alfa and 4 F-PCC in the presence of oral FXa inhibitor-related bleeding.

## Electronic supplementary material

Below is the link to the electronic supplementary material.

Supplementary Material 1

## Data Availability

These analyses were performed using data that are available within the US Department of Veterans Affairs secure research environment, the VA Informatics and Computing Infrastructure (VINCI). All relevant data outputs are within the manuscript and related tables.
