# Correction to: Real-world clinical outcomes among US Veterans with oral factor xa inhibitor-related major bleeding treated with andexanet alfa or 4-factor prothrombin complex concentrate

**DOI:** 10.1007/s11239-023-02841-7

**Published:** 2023-06-12

**Authors:** S. Scott Sutton, Joseph Magagnoli, Tammy H. Cummings, Theresa Dettling, Belinda Lovelace, Mary J. Christoph, James W. Hardin

**Affiliations:** 1 Dorn Research Institute, Columbia VA Healthcare System (151), 6439 Garners Ferry Road, Columbia, SC 29209 USA; 2 Department of Clinical Pharmacy and Outcomes Sciences, College of Pharmacy, University of South Carolina, Columbia, SC USA; 3 Global Health Economics and Outcomes Research, AstraZeneca Rare Disease, Alexion, USA; 4 Department of Epidemiology and Biostatistics, University of South Carolina, 6439 Garners Ferry Road, Columbia, SC 29209 USA

**Correction to: Journal of Thrombosis and Thrombolysis** 10.1007/s11239-023-02820-y

The original publication of this work [1] contained an error in the abstract and text of the manuscript for the clinical and hospitalization outcome. The p-values listed in Table 2 are correct as originally published. The original article has been corrected.

## References

[1] Sutton SS, Magagnoli J, Cummings TH (2023). Real-world clinical outcomes among US Veterans with oral factor xa inhibitor-related major bleeding treated with andexanet alfa or 4-factor prothrombin complex concentrate. J Thromb Thrombolysis.

