# Supplementary material for: Real-world clinical outcomes among US Veterans with oral factor xa inhibitor–related major bleeding treated with andexanet alfa or 4-factor prothrombin complex concentrate
Source: J Thromb Thrombolysis. 2023 May 23;56(1):137–46. doi: 10.1007/s11239-023-02820-y (PMC10284962; doi:10.1007/s11239-023-02820-y)
Supplement: Supplementary file 1 — Supplementary Material 1 [file 11239_2023_2820_MOESM1_ESM.docx]

**Supplementary Information**

**Table S1** ICD-9/10-CM Diagnosis codes and procedure codes for bleeding-related hospitalizations

| **Bleed Type** | **ICD-9 Code** | **ICD-10 Code** | **MSDRG** |
| --- | --- | --- | --- |
| ICH | 430, 431, 432, 432.X,  852.00, 852.01, 852.02, 852.03, 852.05, 852.05, 852.06, 852.09, 852.20, 852.21, 852.22, 852.23, 852.24, 852.25, 852.26, 852.29, 852.40, 852.41, 852.42, 852.43, 852.44, 852.45, 852.46, 852.49, 853.00, 853.01, 853.02, 853.03, 853.04, 853.05  853.06, 853.09, 853.10, 853.11, 853.12, 853.13, 853.14, 853.15, 853.16, 853.19 | I60.%, I61.%, I62.%,  S06.340A, S06.341A, S06.342A, S06.343A, S06.344A, S06.345A, S06.346A, S06.347A, S06.348A, S06.349A, S06.350A, S06.351A, S06.352A, S06.353A, S06.354A, S06.355A, S06.356A, S06.357A, S06.358A, S06.359A, S06.360A, S06.361A, S06.362A, S06.363A, S06.364A, S06.365A, S06.366A, S06.367A, S06.368A, S06.369A, S06.369A, S06.4X0A, S06.4X1A, S06.4X2A, S06.4X3A, S06.4X4A, S06.4X5A, S06.4X6A, S06.4X7A, S06.4X8A, S06.4X9A, S06.5X0A, S06.5X1A, S06.5X2A, S06.5X3A, S06.5X4A, S06.5X5A, S06.5X6A, S06.5X7A, S06.5X8A, S06.5X9A, S06.6X0A, S06.6X1A, S06.6X2A, S06.6X3A, S06.6X4A, S06.6X5A, S06.6X6A, S06.6X7A, S06.6X8A, S06.6X9A | 023, 023, 082, 083, 084, 085, 086, 087, 955, 956, 957, 958, 959, 963, 964, 965 |
| GI | 531.0x, 531.2x, 531.4x, 531.6x, 532.0x, 532.2x, 532.4x, 532.6x, 533.0x, 533.2x, 533.4x, 533.6x, 534.0x, 534.2x, 534.4x, 534.6x, 535.01, 535.11, 535.21, 535.31, 535.41, 535.51, 535.61, 537.83, 456.2, 530.7, 530.82, 578.X, 455.2, 455.5, 455.8, 562.02, 562.03, 562.12, 562.13, 568.81, 569.3, 569.85 | K25.0, K25.2, K25.4, K25.6, K26.0, K26.2, K26.4, K26.6, K27.0, K27.2, K27.4, K27.6, K28.0, K28.2, K28.4, K28.6, K29.01, K29.21, K29.31, K29.41, K29.51, K29.61, K29.71, K29.81, K29.91, K31.811, I85.01  I85.11, K22.6, K22.8, K92.0, K55.21, K57.01, K57.11, K57.13, K57.21, K57.31, K57.33, K57.41, K57.51, K57.53, K57.81, K57.91, K57.93, K62.5, K64.0, K64.1, K64.2, K64.3, K64.4, K64.8, K92.1, K92.2 | 377, 378, 379, 812, 391, 392, 368, 369, 370, 380, 381, 382, 393, 394, 395 |
| Other bleeds (non-ICH, non-GI) | 423.0, 459.0, 568.81, 719.1x, 784.7, 784.8, 786.3, 620.7, 376.32, 364.41, 363.61, 363.62, 363.63, 363.72, 362.81, 362.43, 379.23, 360.43, 377.42, 380.31, 593.81, 599.70, 626.2, 626.8, 626.4, 626.7, 626.9, 599.71, 599.70 | D68.32, T45.515A, T45.515S, T45.515D, I31.2, R58, K66.1, M25.%, R04.0, R04.1, R04.2, R04.9, R04.81, R04.89, H05.239, H21.03, H31.309, H31.319, H31.329, H31.419, H35.60, H35.739, H43.13, H44.819, H47.029, H61.129, N83.7, N89.7, N28.0, N92.0, N92.1, N92.5, N93.0, N93.8, N93.9, R31.0, R31.9 | 813, 917, 918, 555, 556, 204, 150, 151, 011, 012, 013, 154, 155, 156 |

**Table S2** Propensity Score–weighted Sample Characteristics

| ***Baseline characteristics*** | ***Andexanet alfa*** | ***4F-PCC*** | ***Standardized difference*** |  |
| --- | --- | --- | --- | --- |
|  |  |  |  |  |
| Age | 74.6 | 73.5 | 0.094 |  |
| Race |  |  |  |  |
| *Black* | 17% | 20% | 0.078 |  |
| *Other/unknown* | 5% | 5% | 0.043 |  |
| *White* | 79% | 75% | 0.093 |  |
| Sex |  |  |  |  |
| *Female* | 0% | 2% | 0.123 |  |
| *Male* | 100% | 99% | 0.123 |  |
| Charlson comorbidity Index | 5.551 | 5.472 | 0.024 |  |
| Bleed type |  |  |  |  |
| *GI* | 48% | 51% | 0.07 |  |
| *ICH* | 28% | 28% | 0.001 |  |
| *Other* | 25% | 21% | 0.088 |  |
| Anticoagulant |  |  |  |  |
| *Apixaban* | 71% | 59% | 0.24 |  |
| *Edoxaban* | 1% | 0% | 0.079 |  |
| *Enoxaparin* | 8% | 25% | 0.39 |  |
| *Rivaroxaban* | 20% | 16% | 0.126 |  |
| Ventilation | 21% | 28% | 0.16 |  |
| Invasive ventilation | 15% | 16% | 0.043 |  |
| Concomitant Medications |  |  |  |  |
| *Plasma* | 11% | 14% | 0.105 |  |
| *Cryoprecipitates* | 1% | 4% | 0.15 |  |
| *Transfusion* | 10% | 12% | 0.083 |  |
| *Tranexamic acid* | 1% | 1% | 0.05 |  |
| *Vitamin K* | 16% | 32% | 0.328 |  |
| *Platelets* | 11% | 13% | 0.055 |  |
| *Red blood cells* | 34% | 36% | 0.05 |  |
| *Activated 4F-PCC* | 5% | 5% | 0.016 |  |

4F-PCC = four-factor prothrombin complex concentrate, GI = gastrointestinal; ICH = intracranial hemorrhage.

**Table S3** Length of Stay Ratios: Gamma Generalized Linear Model, Log Link

|  | ***Length of Stay*** | ***PS-weighted Length of Stay*** |
| --- | --- | --- |
| ***Full cohort*** | ***LOS ratio (95% CI)*** | ***LOS ratio (95% CI)*** |
| Intercept | 6.28 (1.39-40.93) | 7.53 (2.58-21.95) |
| Andexanet alfa vs. 4F-PCC (ref) | 0.85 (0.6-1.21) | 0.88 (0.64-1.23) |
| Age | 1 (0.98-1.01) | 0.99 (0.98-1.01) |
| White vs. non-white (ref) | 0.85 (0.57-1.24) | 0.9 (0.63-1.29) |
| Male vs. Female (ref) | 2.46 (0.51-7.4) | 2.16 (0.93-5.03) |
| Charlson Comorbidity Index | 1.01 (0.96-1.06) | 1 (0.96-1.05) |
| Bleed type: ICH vs. GI (ref) | 1.22 (0.84-1.8) | 1.25 (0.84-1.88) |
| Bleed type: other vs. GI (ref) | 1.82 (1.19-2.86) | 1.47 (0.99-2.2) |
| Ventilation vs. no ventilation (ref) | 1.31 (0.89-1.97) | 1.09 (0.73-1.64) |
| Transfusion vs. no transfusion (ref) | 0.93 (0.58-1.55) | 0.98 (0.62-1.54) |
| ***Cohort surviving hospitalization*** | ***Length of Stay*** | ***PS-weighted Length of Stay*** |
|  | ***LOS ratio (95% CI)*** | ***LOS ratio (95% CI)*** |
| Intercept | 9.33 (1.31-122.64) | 10.77 (2.83-40.93) |
| Andexanet alfa vs. 4F-PCC (ref) | 0.72 (0.51-1.03) | 0.76 (0.54-1.06) |
| Age | 1 (0.98-1.01) | 0.99 (0.98-1.01) |
| White vs. non-white (ref) | 0.92 (0.61-1.36) | 0.96 (0.65-1.42) |
| Male vs. Female (ref) | 1.64 (0.17-6.39) | 1.42 (0.55-3.68) |
| Charlson Comorbidity Index | 1.01 (0.96-1.07) | 1 (0.96-1.05) |
| Bleed type: ICH vs. GI (ref) | 1.31 (0.88-1.98) | 1.36 (0.9-2.05) |
| Bleed type: other vs. GI (ref) | 1.6 (1.04-2.52) | 1.35 (0.88-2.06) |
| Ventilation vs. no ventilation (ref) | 1.66 (1.07-2.66) | 1.33 (0.8-2.23) |
| Transfusion vs. no transfusion (ref) | 1.15 (0.66-2.16) | 1.19 (0.69-2.06) |

4F-PCC = four-factor prothrombin complex concentrate, CI = confidence interval, GI = gastrointestinal, ICH = intracranial hemorrhage, LOS = length of stay, PS = propensity-score, ref = reference group.

**Figure S1** IPTW Kaplan-Meier survival curves: in-hospital mortality


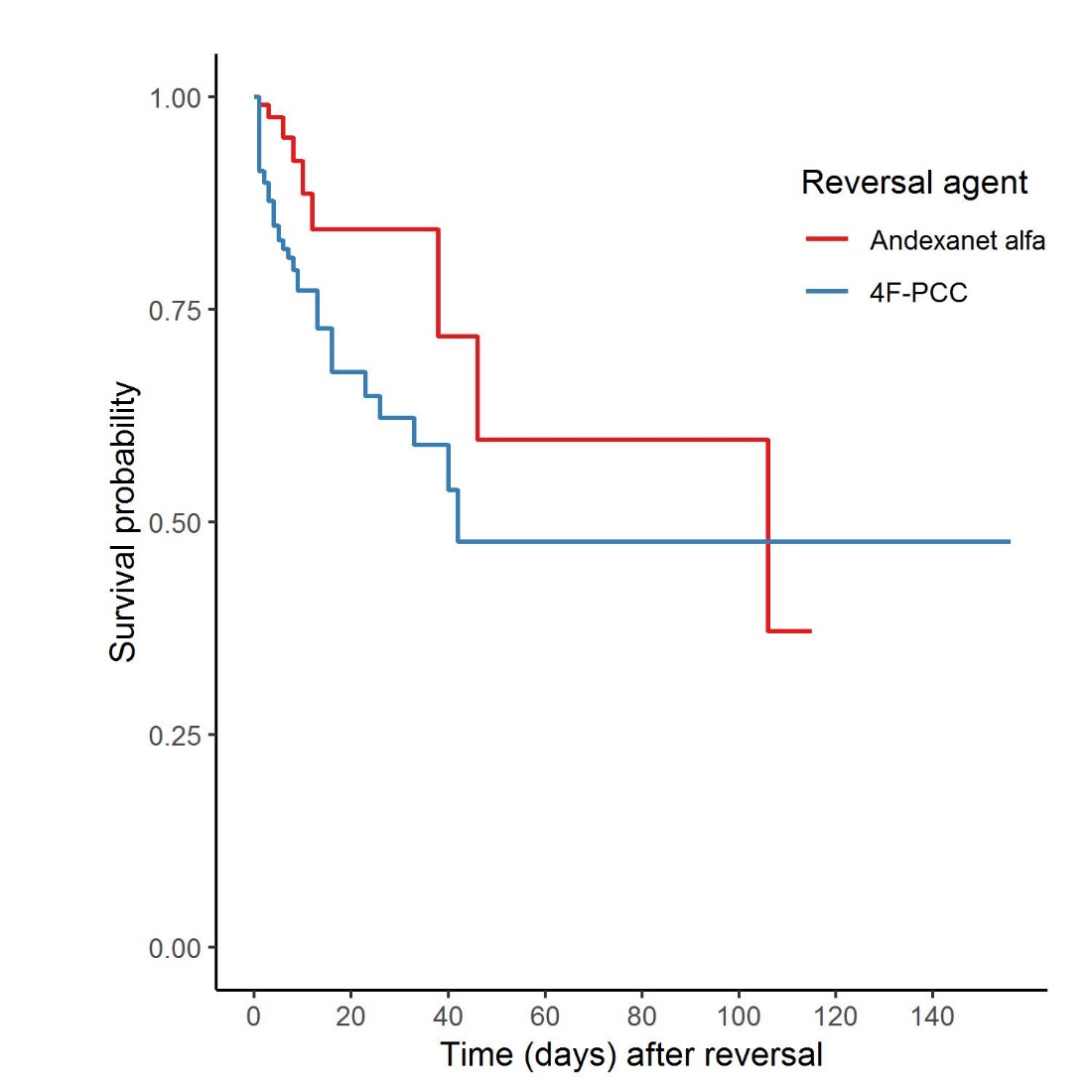


4F-PCC = 4-factor prothrombin complex concentrate

**Figure S2** IPTW Kaplan-Meier survival curves: 30-day mortality


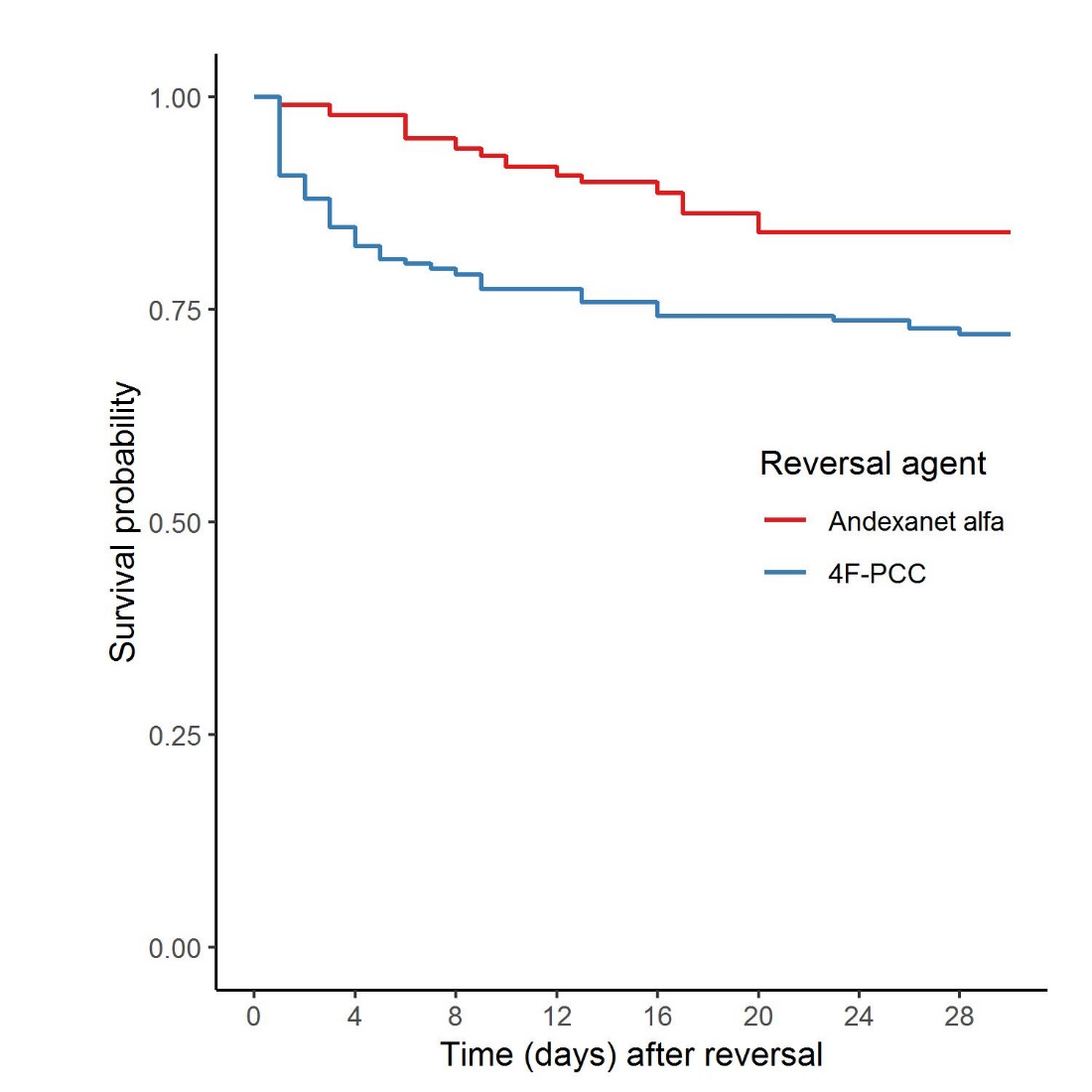


4F-PCC = 4-factor prothrombin complex concentrate

**Figure S3** Standardized differences before and after IPTW

**
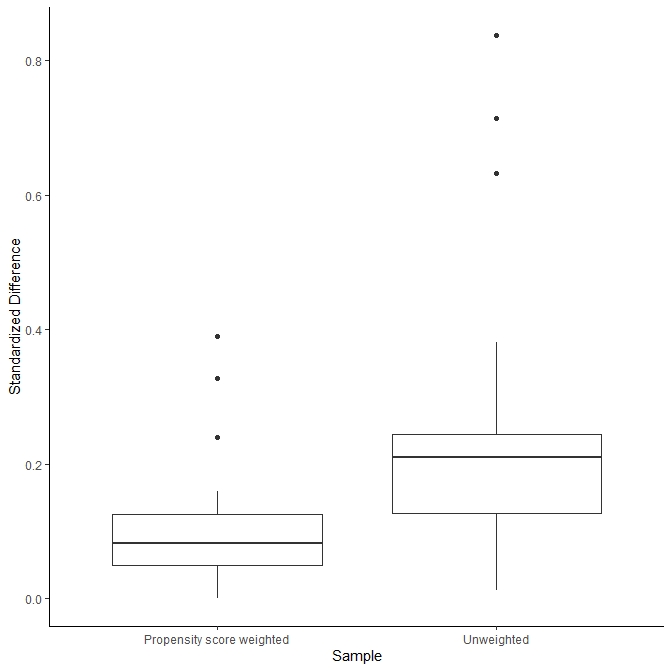
**

**Table S4** IPTW weighted accelerated failure time models (AFT) for in-hospital mortality

|  | ***Weibull*** | ***Exponential*** | ***log-normal*** | ***log-logistic*** |
| --- | --- | --- | --- | --- |
| ***Variable*** | ***e ^β_hat^ (95%CI)*** | ***e ^β_hat^ (95%CI)*** | ***e ^β_hat^ (95%CI)*** | ***e ^β_hat^ (95%CI)*** |
| Intercept | 115.58 (3.6-3711.51) | 85.74 (3.86-1906.48) | 68.95 (4.45-1067.31) | 74.13 (3.48-1578.81) |
| Andexanet alfa vs. 4F-PCC (ref) | 4.11 (1.34-12.6) | 3.44 (1.3-9.05) | 3.35 (1.42-7.92) | 3.3 (1.23-8.82) |
| Age | 0.98 (0.94-1.02) | 0.98 (0.94-1.01) | 0.99 (0.95-1.02) | 0.98 (0.94-1.02) |
| White vs. non-white (ref) | 0.56 (0.21-1.5) | 0.6 (0.26-1.41) | 0.57 (0.24-1.38) | 0.53 (0.19-1.43) |
| Male vs. Female (ref) | 24.02 (4.19-137.85) | 16.94 (3.46-82.9) | 14.32 (2.91-70.44) | 20.51 (4.23-99.34) |
| Charlson Comorbidity Index | 0.85 (0.77-0.93) | 0.87 (0.81-0.95) | 0.85 (0.78-0.92) | 0.83 (0.76-0.91) |
| Bleed type: ICH vs. GI (ref) | 0.86 (0.31-2.38) | 0.98 (0.41-2.37) | 0.68 (0.28-1.64) | 0.73 (0.27-1.94) |
| Bleed type: other vs. GI (ref) | 2.17 (0.84-5.61) | 1.98 (0.88-4.42) | 2.56 (1.07-6.12) | 2.86 (1.06-7.72) |
| Ventilation vs. no ventilation (ref) | 0.21 (0.08-0.58) | 0.28 (0.12-0.64) | 0.18 (0.08-0.41) | 0.18 (0.07-0.48) |
| Transfusion vs. no transfusion (ref) | 0.49 (0.19-1.31) | 0.53 (0.23-1.24) | 0.46 (0.18-1.17) | 0.49 (0.16-1.54) |
